# Lessons Learned From Enhancing Vaccine Pharmacovigilance Activities During PsA-TT Introduction in African Countries, 2010–2013

**DOI:** 10.1093/cid/civ599

**Published:** 2015-11-09

**Authors:** Fabien V. K. Diomandé, Téné M. Yaméogo, Kirsten S. Vannice, Marie-Pierre Preziosi, Simonetta Viviani, Claude-Roger Ouandaogo, Modibo Keita, Mamoudou H. Djingarey, Nehemie Mbakuliyemo, Bartholomew Dicky Akanmori, Samba O. Sow, Patrick L. F. Zuber

**Affiliations:** 1 Centers for Disease Control and Prevention, Atlanta, Georgia; 2 Institut Supérieur des Sciences de la Santé, Université Polytechnique de Bobo-Dioulasso, Burkina Faso; 3 Department of Immunization, Vaccines and Biologicals, World Health Organization, Geneva, Switzerland; 4 Meningitis Vaccine Project, PATH, Ferney-Voltaire, France; 5 Meningitis Vaccine Project, Department of Immunization, Vaccines and Biologicals, World Health Organization, Geneva, Switzerland; 6 Direction Générale de la Pharmacie du Médicament et des Laboratoire, Ministère de la santé, Ouagadougou, Burkina Faso; 7 Centre pour le Développement des Vaccins, Ministère de la Santé, Bamako, Mali; 8 Inter-country Support Team for West Africa, World Health Organization, Ouagadougou, Burkina Faso; 9 Department of Immunization Vaccines and Emergency, World Health Organization, Regional Office for Africa, Brazzaville, Republic of Congo; 10 Department of Essential Medicines and Health Products, World Health Organization, Geneva, Switzerland

**Keywords:** group A meningococcal vaccine, PsA-TT, AEFI, pharmacovigilance systems, African meningitis belt

## Abstract

***Background.*** The rollout of the group A meningococcal vaccine, PsA-TT, in Africa's meningitis belt countries represented the first introduction of a vaccine specifically designed for this part of the world. During the first year alone, the number of people who received the vaccine through mass vaccination campaigns was several hundredfold higher than that of subjects who participated in the closely monitored clinical trials. Implementation of a system to identify rare but potentially serious vaccine reactions was therefore a high priority in the design and implementation of those campaigns.

***Methods.*** National authorities and their technical partners set up effective vaccine pharmacovigilance systems, including conducting active surveillance projects.

***Results.*** Implementation of national expert advisory groups to review serious adverse events following immunization in all countries and active monitoring of conditions of interest in 3 early-adopter countries did not identify particular concerns with the safety profile of PsA-TT, which had already provided tremendous public health benefits.

***Conclusions.*** Lessons learned from this experience will help to improve preparations for future vaccine introductions in resource-poor settings and capitalize on such efforts to advance vaccine safety systems in the future.

A group A meningococcal conjugate vaccine, PsA-TT, was developed by the Meningitis Vaccine Project and the Serum Institute of India, Ltd (SIIL). Clinical trials conducted among approximately 10 000 persons aged 1–29 years, in India and in Africa, confirmed a vaccine safety profile similar to that of licensed polysaccharide vaccines, as well as a stronger and more persistent immunological response against group A meningococcus (MenA) [1, 2]. The vaccine was licensed by the Drug Controller General of India in January 2010 and prequalified by the World Health Organization (WHO) in June 2010, and a common regulatory dossier was submitted to the first 3 countries (Burkina Faso, Mali, and Niger) by SIIL prior to introducing the vaccine , in accordance with regulatory requirements. The other countries that have used this vaccine in mass campaigns received summary lot protocols and used an expedited review procedure to license the vaccine [3, 4]. By 2013, 12 countries in the epidemic meningitis belt had introduced PsA-TT, vaccinating >150 million eligible people. As a result, a significant decrease in MenA carriage was documented among vaccinated and unvaccinated individuals [5], and the burden of meningitis from MenA dramatically decreased [6, 7].

Clinical trials are limited in their ability to detect rare and late-occurring adverse events associated with vaccination due to small sample sizes and limited participant follow-up time [8–10]. Careful monitoring for possible infrequent but serious adverse events following immunization (AEFIs) is thus desirable during postmarketing surveillance and widespread use of any vaccine. The Global Advisory Committee on Vaccine Safety (GACVS), during its session in December 2009, recommended an initial phased approach to introduction of PsA-TT to carefully collect additional safety data [11]. Postmarketing surveillance for AEFIs after PsA-TT mass immunization campaigns was systematically conducted in all countries that used the vaccine [12, 13]. This served as an opportunity for capacity building and to conduct postlicensure safety surveillance in the low- and middle-income countries of Africa where the vaccine was used.

Conducting postmarketing surveillance for AEFIs after PsA-TT introduction in these resource-constrained countries highlighted several challenges. This article describes how pharmacovigilance systems were set up in selected countries from 2010 to 2013, discusses the main constraints and challenges faced, and analyzes lessons learned.

## PSA-TT PHARMACOVIGILANCE: OBJECTIVES, METHODS, AND RESULTS

PsA-TT was introduced through 10-day mass vaccination campaigns targeting individuals 1–29 years old, typically representing about 70% of a country's total population. Criteria for the selection of countries to be among the first to introduce PsA-TT were (1) presence of epidemic risk indicators; (2) presence of case burden indicators; (3) size of the target population; (4) expected annual vaccine supply; and (5) participation of population in clinical trials of PsA-TT. Based on these criteria, PsA-TT was first introduced in Burkina Faso (2010), Mali (2010–2011), and Niger (2010–2011), followed by Chad (2011–2012), Cameroon (2011–2012), Nigeria (2011–2014), Ghana (2012), Benin (2012), Senegal (2012), Sudan (2012), The Gambia (2013), and Ethiopia (2013).

The postmarketing safety surveillance of PsA-TT focused on reporting all AEFIs during the 10 days of the mass vaccination campaigns, and up to 42 days thereafter to capture late-onset adverse events. This surveillance allowed for initiating proper clinical management of reported AEFI cases, estimating reported incidence rates, investigating and classifying all serious AEFIs through causality assessment, and strengthening national vaccine safety monitoring systems.

**Figure 1.**
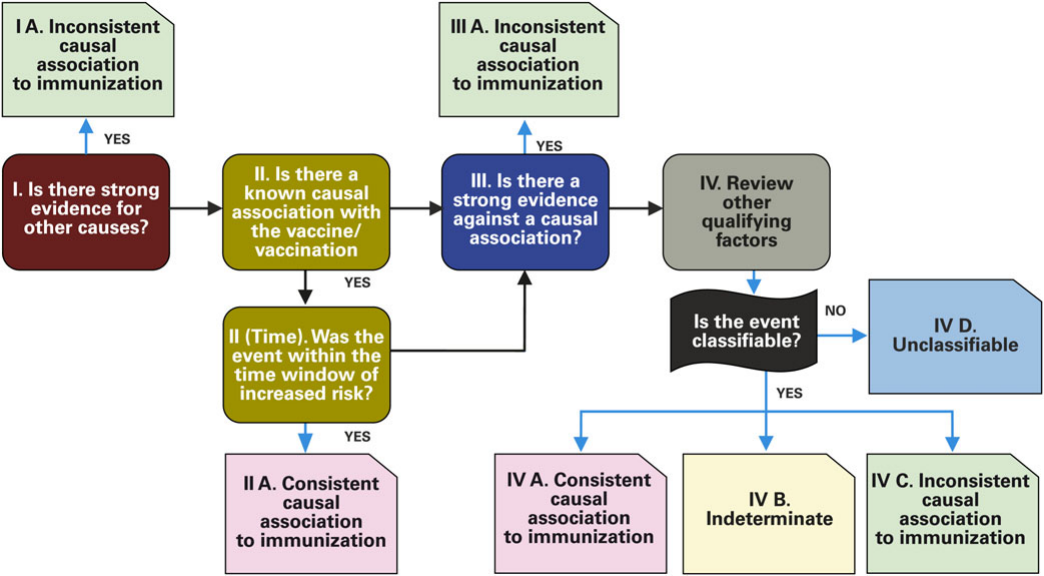
PsA-TT causality assessment scheme for an individual case that meets a case definition. Source: World Health Organization [14].

Vaccine safety surveillance was built upon existing national AEFI surveillance systems. In all countries, the necessary procedures, tools, and terms of reference were reviewed or established (Table 1). The tools for surveillance included field guides, case definitions, case-based notification forms, case investigation forms for serious AEFIs, and job aids; these were made available at all levels of the healthcare system. Initial protocols and guidance documents were reviewed and approved by GACVS in December 2009, prior to country implementation. National Expert Committees (NECs) for AEFI causality assessments were established in each country. Prior to the first introduction of PsA-TT, members of the NECs from Burkina Faso, Mali, and Niger gathered in Ouagadougou, Burkina Faso, and reviewed the proposed procedures and tools during a 4-day workshop. In addition, the new WHO method for AEFI causality assessment was presented (Figure 1) [14]. The NEC members subsequently reviewed their national guidelines and tools for PsA-TT safety surveillance and trained their healthcare workers and other staff involved (immunization and surveillance officers and community workers). National experts from other countries were similarly trained.

**Table 1.** Panel of Documents and Tools for Group A Meningococcal Conjugate Vaccine (PsA-TT) Pharmacovigilance

Protocol and Standard Operating Procedures	Tools	Resource Groups (With Terms of Reference)
Standard operating procedures for AEFI monitoring Case definitions Active surveillance protocol Anaphylaxis management	Notification form Investigation form Active search form Weekly summary sheet Biological samples shipment sheet Autopsy consent form Supervision form	National Experts Committee AEFI central team Reference laboratory AEFI focal persons Regional AEFI team District AEFI team Hospital AEFI team Health facility AEFI team

Abbreviation: AEFI, adverse events following immunization.

All serious AEFI cases reported were reviewed by the appropriate NECs, which could request further clinical and/or laboratory investigations as needed. A final classification was provided for each severe AEFI case according to WHO criteria: consistent causal association (which has a further classification of possibly vaccine-related or probably vaccine-related), inconsistent, unclassifiable, or indeterminate. Country coordination teams at central (national), regional, and district levels were designated and trained on their tasks, roles, and responsibilities according to previously developed standard operating procedures (SOPs) (Table 2). These teams played a key role in managing logistics to support the functioning of the overall system, and in ensuring proper monitoring of surveillance activities and supervision. The central coordination teams ensured that the NECs received complete, good-quality data on each severe AEFI case reported for causality assessment; healthcare workers were trained on procedures of identification and notification of AEFIs; and populations, healthcare workers, and community workers were sensitized on AEFI reporting and notification.

**Table 2.** Country Coordination and Roles and Responsibilities Scheme

Level of the Health System	Team	Tasks
Central	National Expert Committee	Investigation and classification
	Central AEFI Team	Coordination, supervision, investigation, data management
	Reference laboratory (and focal person)	Confirmation of laboratory testing, specimen storage
	Reference hospital (and focal person)	Confirmation of clinical diagnosis and cases management
Regional	Reference hospital (and focal person)	Confirmation of clinical diagnosis and cases management
	Regional medical team	Support district team for coordination and supervision
District	District AEFI team	Coordination, supervision, investigation, data management
Health facilities	Nurses and vaccinators	Detection, notification, case management

Abbreviation: AEFI, adverse events following immunization.

Active surveillance was conducted as recommended by GACVS to improve monitoring for specific AEFIs [12]. Enhanced AEFI sentinel surveillance during the 2010 campaigns in Burkina Faso (Ziniaré health district) and in Mali (Commune VI of Bamako health district) consisted of active solicitation of AEFI reports for 12 preselected adverse events easily recognizable at the peripheral health facility level. This enhanced surveillance required substantial efforts to simplify reporting forms so they could be managed by district staff. Also, job aids were prepared with simplified case definitions and SOPs, and supervision of activities was improved. In Burkina Faso, a retrospective registry review of outcomes of interest was conducted during and immediately after the vaccination campaign in community clinics. This allowed description of the pattern of clinical diagnoses usually seen in the population under surveillance (ie, background rates) and assessment of any changes temporally associated with the vaccination campaigns. The respective frequencies of health problems reported were described and their occurrences analyzed according to vaccination periods [12].

The initial analyses from passive and active surveillance efforts during the first mass campaigns were presented to GACVS during its session of June 2011, and surveillance approaches and guidelines were subsequently adapted through simplification of procedures, job aids, and sensitization of healthcare workers. After each vaccination campaign, a comprehensive country report describing methods and results of AEFI monitoring was submitted to the vaccine manufacturer.

In the most intensive active surveillance study conducted, prespecified outcomes during the November 2011 campaign in Mali were identified actively through data routinely collected in community clinic registers matched to self-reported vaccination status [15]. A population of approximately 250 000 in the indicated age range was under active surveillance in vaccinated districts, and clinic visits for any reason were recorded by an independent data entry team. This approach was chosen to alleviate the burden of AEFI reporting on clinicians, to integrate the surveillance system with the routine health system, to systematically collect cases regardless of vaccination status, to allow for the possibility of detecting unanticipated AEFIs, and to more closely approximate incidence rates in vaccinated and unvaccinated groups.

A regional coordination team—consisting of experts from the WHO Inter-country Support Team for West Africa and WHO headquarters as well as selected experts and consultants from African countries—provided technical support to countries introducing PsA-TT regarding all the components of the planning and implementation of AEFI surveillance systems. In each country introducing PsA-TT, vaccine safety monitoring activities were funded as part of the overall mass vaccination campaign.

From 2010 to 2013, the monitoring of AEFIs through enhanced passive and sentinel active surveillance did not identify any outstanding safety issues [12, 13]. Among people vaccinated, 24 841 cases of AEFIs were reported through the newly established enhanced passive surveillance systems (Table 3). There were 24 398 mild and 443 serious AEFIs, with country-level reporting rates of serious events ranging from 0.00 to 0.52 per 100 000 persons vaccinated. The assessment of serious cases by countries' NECs did not identify a consistent causal relationship between the vaccine and the occurrence of observed events [12, 13]. However, many possible vaccine-related hypersensitivity reactions (eg, urticaria, bronchospasm) were reported [12, 13]. Active surveillance in Mali identified an increased risk of fever following vaccination, although no corresponding increase in the risk of febrile seizures was seen [15]. Continued safety monitoring of PsA-TT during its rollout should further assess any association of vaccine components with anaphylactic reactions.

**Table 3.** Adverse Events Following Immunization Reported by Country

Country	Year of PsA-TT Vaccination	Population Vaccinated, No.	AEFIs Reported			
			Total, No.	Mild, No.	Serious, No.	Reporting Rates of Serious AEFIs^a^
Burkina Faso	2010	11 421 502	1890	1857	33	0.29
Mali	2010, 2011	11 109 484	573	538	35	0.32
Niger	2010, 2011	10 575 365	534	486	48	0.45
Chad	2011, 2012	8 724 890	3703	3657	46	0.53
Cameroon	2011, 2012	6 108 772	836	817	19	0.31
Nigeria	2011, 2012, 2013	51 745 045	15 209	15 137	72	0.14
Ghana	2012	3 038 393	555	555	0	0
Benin	2012	2 718 459	177	173	4	0.15
Senegal	2012	4 216 691	160	151	9	0.21
Sudan	2012, 2013	23 586 086	704	670	34	0.14
The Gambia	2013	1 229 509	8	8	0	0
Ethiopia	2013	18 625 074	492	349	143	0.77
Total	2010–2013	153 099 270	24 841	24 398	443	0.29

Abbreviation: AEFI, adverse events following immunization.

^a^ No. of serious AEFI cases reported per 100 000 persons vaccinated.

## CONSTRAINTS AND CHALLENGES FACED

### Planning and Preparedness

The first countries to introduce PsA-TT (Burkina Faso, Mali, and Niger) received sufficient resources for planning and implementing effective pharmacovigilance systems. They based their approach on existing passive AEFI surveillance and implemented, at limited scale, active search and surveillance of preselected events deemed of interest because there was a theoretical possibility for a relationship with vaccination. Unfortunately, after these initial intensive pharmacovigilance activities, resources allocated to AEFI monitoring decreased over time, with negative consequences on the quality of the safety data collected. Even though AEFI surveillance was still included in national PsA-TT implementation plans, activities were mostly limited to the integrated training of immunization officers, with very limited resources available for monitoring and supervision. In addition, there were almost no resources to set up NECs for review of serious AEFIs and to support their work. As a result, subsequent PsA-TT campaigns did not benefit from the same attention to AEFI surveillance. This resulted in inadequate application of standard case definitions, lower-quality and less-complete data reported, underreporting of cases, limited country reports on PsA-TT pharmacovigilance activities, and detrimental delays in submission of those reports to the vaccine manufacturer. In addition, insufficient planning led to important delays in the startup of activities. In Mali, the decision to conduct a special active surveillance study was made 4 months prior to the campaign, leaving limited time for planning, particularly recruiting and training a data collection team, developing data entry screens and databases, pilot-testing data entry forms, and sensitizing clinicians to obtain the vaccination status of patients.

### Defining Roles and Responsibilities

In most countries that have introduced PsA-TT, AEFI monitoring was previously vested with the Expanded Programme on Immunization (EPI). The planning and implementation of AEFI monitoring requires close collaboration between various health programs in the same country: the primary healthcare system, EPI, national regulatory authority (NRA) for health products, and, where they exist, pharmacovigilance centers. This need for collaboration was associated with many constraints and challenges in finalizing workplans and budgets, sharing of roles and responsibilities, and effectively monitoring activities and supervising healthcare workers involved with AEFI monitoring.

AEFI passive surveillance during the 10 days of the mass vaccination campaign was the responsibility of the EPI, but the NRA was responsible for AEFI monitoring during the subsequent 42 days. This dual leadership for the same activity caused many difficulties in the allocation of resources and coordination of activities. Indeed, in most instances, financial resources were almost entirely allocated to EPI, leaving few resources for the vaccine regulatory authority to monitor late-onset AEFIs. This disparity in resource distribution had a significant impact on the detection and reporting of AEFIs. In addition, many program partners and stakeholders (vaccinators, national and international supervisors, etc) were present in the field to improve AEFI detection and notification rates during the mass vaccination campaign, whereas their involvement was minimal during the subsequent 42 days. The drop of resources after the campaign end to monitor late-onset AEFIs could be the reason that that 70% of AEFI cases were reported during the 10-day campaign and 96% during the first 16 days following the start of the campaign [12, 13].

### Establishing National Expert Committees for Causality Assessment

Establishing NECs for PsA-TT AEFI causality assessments was a key step toward building the capacity of countries' pharmacovigilance systems. NECs played an important role in training national actors involved in AEFI monitoring and in having an oversight function for the entire system. The major challenge was to secure sufficient funds after the initial phase of vaccine introduction in 2010–2011 to enable NECs to be fully functional (eg, payment of per diems, logistics for meetings, and cases investigation) during the whole surveillance period.

Because of the lack of resources in many countries during the later vaccine rollout phase, AEFI cases reported were not appropriately reviewed, and investigations and causality assessments were not appropriately conducted for severe AEFIs cases reported.

### Guidelines and Tools Development and Staff Training

The weaknesses of existing vaccine pharmacovigilance systems in countries introducing PsA-TT, and the limited experience with enhanced, active AEFI monitoring in low-resource settings, required developing new guidelines, SOPs, and many new tools to train program staff. In the case of active surveillance, new surveillance designs were pilot-tested and new analytical methods were applied for the first time in these settings. Also, integrating new guidelines and tools for vaccine pharmacovigilance into existing EPI frameworks was a challenge. Establishing a platform for collaboration between the primary healthcare system, EPI, and NRAs for health products was a challenge in most countries.

### Understanding Local Clinical Standards to Set up Sensitive Detection of Adverse Events and Adequately Record Them

The active surveillance system established in Mali used routinely collected health center register data. As a result, the level of clinical case information and the specificity of the diagnoses were limited. This approach, however, allowed access to comprehensive information from the existing health system to monitor a larger population without placing any additional burden on health center staff. Safety monitoring would be greatly enhanced with improved general diagnostic capacity at the clinic level and with data capture systems, such as electronic records, that would facilitate analysis of large numbers of records in a timely fashion.

### Knowing Background Rates of Conditions of Interest

Background rates for many outcomes vary by year and setting [16]. Selecting the proper comparison rate (eg, historical rates or rates from a comparator population) therefore involves potential uncertainties. Seasonality of infectious diseases that may cause many clinical symptoms of interest, such as fever or convulsions, is particularly problematic for applying statistical methods, such as self-controlled case series, during mass vaccination campaigns when all vaccinations are clustered in a small point in time. In addition, complex serious adverse events of interest, such as Guillain-Barré syndrome, are not diagnosed as such in primary clinic settings, where acute flaccid paralysis will likely be recorded. Use of existing data for estimation of background rates therefore needs to be tailored to local clinical standards.

### Documentation of Vaccination Status

Collecting information on the vaccination status of persons with an AEFI is key to causality assessment. Needed information includes the name and date of administration of the primary suspected vaccine, the names and dates of other vaccines given prior to the AEFI, the batch (lot) number of all vaccines received, and the dose number [17]. During preparations for PsA-TT campaigns, all countries planned to make vaccination cards available at all vaccination posts, and all those vaccinated were to receive these cards as proof of vaccination. In addition, health centers and hospitals were asked to record (in their consultation registers) the PsA-TT vaccination status of all patients presenting to these facilities after the start of the campaign.

There were many difficulties, however, in the use of immunization cards and in documenting immunization status in facility registers. In some cases, there were insufficient or no vaccination cards available in several districts or even entire regions, leading to large disparities across regions in the collection of information on vaccination status. Also, even when cards were available, they were sometimes incompleted or incorrectly maintained.

In Burkina Faso, during active AEFI surveillance in the district of Ziniaré, the vaccination status of people with 12 clinical conditions that could be related to vaccination was verified through the review of vaccination cards when available, registers of consultation at health posts, or verbal reports by parents. For active surveillance studies in Mali, vaccination status was determined by parental reporting, which is known to be unreliable. In the absence of written information on vaccination status, causality assessment was based on verbal reporting.

The observed shortcomings in establishing the vaccination status among AEFI cases have implications beyond the issue of causality assessment. Poor documentation is a major constraint for vaccine coverage surveys and for the implementation and analysis of data from special studies to assess the impact of new vaccines.

### Use of PsA-TT Among Pregnant and Lactating Women

Because PsA-TT campaigns involved people 1–29 years of age, a significant proportion of eligible vaccine recipients were pregnant or lactating women. In November 2010, WHO issued a technical note recommending that pregnant and lactating women residing in the meningitis belt receive PsA-TT during any stage of pregnancy or lactation [18]. This recommendation was based on the knowledge that the benefits of vaccination would far outweigh any potential risk. Despite this recommendation, however, several national authorities were reluctant to allow vaccination of pregnant and lactating women and brought the issue to WHO's attention during campaign planning. This concern was due in part to the vaccine manufacturer's statement on the package insert: “Meningococcal A conjugate vaccine is not recommended in pregnancy unless there is a definite risk of group A meningococcal disease” [19].

Most countries, however, followed the WHO recommendation and gave clear instructions to their EPI programs and healthcare workers to use PsA-TT in pregnant and lactating women. In general, the risk of MenA disease is well understood, which is consistent with the statement on the package insert. So far, available safety surveillance reports have not identified specific risks to the mother or fetus related to PsA-TT administration during pregnancy and lactation. Studies focusing specifically on pregnant women are difficult to design because of limited use of antenatal services in most vaccinated populations, especially during the early stages of pregnancy. Approaches using demographic health surveys from vaccination sites are currently being explored.

## CONCLUSIONS AND RECOMMENDATIONS

Safety monitoring of vaccination with PsA-TT did not identify major concerns related to use of the vaccine. Most of the reassuring data were generated during the initial rollouts in 2010–2011. Differences in national AEFI reporting rates can be largely attributed to the amount of time, energy, and resources made available for monitoring. The high profile PsA-TT program offered a unique opportunity to pilot active surveillance systems in Burkina Faso and Mali, through which many lessons were learned about challenges and opportunities.

The extensive efforts to plan and implement the PsA-TT campaigns contrast with the limited attention traditionally given to vaccine safety monitoring during routine vaccination. The PsA-TT pharmacovigilance approach focused on setting up or reinforcing a basic national system to collect data, raising awareness on vaccine safety issues during the campaign (including crisis communication), and establishing an NEC for causality assessment during and immediately after the mass vaccination campaign. Although the capacities developed will theoretically remain available after the PsA-TT mass campaigns, this work will likely have little long-term impact on the capacity of NRAs to maintain a pharmacovigilance system to monitor routine immunization programs. Indeed, none of these countries fulfill the requirements for a functional NRA [20]. National systems for detecting and managing adverse drug and vaccine reactions are weak, and in most cases there is no spontaneous reporting system. In addition, there is no stable funding for ongoing operation of NECs, which are unable to meet on a regular basis; existing NRAs do not have stable basic funding, clear mandates, and enough designated staff for pharmacovigilance; and there is no clear country communication strategy to address issues of vaccine safety for routine programs.

Based on experiences with PsA-TT vaccine safety monitoring during mass vaccination campaigns, the authors propose the following recommendations for future vaccine safety activities in low-income settings:

- At the global level, advocacy should be conducted to mobilize more resources for capacity-building of national vaccine pharmacovigilance systems, as proposed in the Global Vaccine Safety Blueprint [21]. Special attention should be given to reinforce NRAs' capacities to support vaccine pharmacovigilance during both mass vaccination campaigns and routine immunizations. The timely availability of sufficient resources is critical to ensure high-performing, sustainable pharmacovigilance systems.
- Early identification of countries where a new vaccine will be introduced is desirable for proper implementation of active surveillance, including measurement of background rates of conditions of interest in advance of vaccine introduction. Conversely, readiness for active surveillance could be one criterion for selecting early introducers.
- At the country level, institutional coordination between the EPI and the vaccines regulatory authority is required for planning and implementation of vaccine pharmacovigilance systems. This should be accomplished by clarifying roles and responsibilities in national vaccine safety surveillance guidelines and paying more attention to resource distribution.
- Documentation of the immunization status of AEFI cases should be considered a high priority during the planning and implementation of vaccination campaigns and routine immunization. Financial and logistical resources must be available in time for the procurement and distribution of vaccination cards. Healthcare workers should be trained to complete the cards and document immunization status in consultation registers during the AEFI surveillance period. Populations and communities must also be sensitized to the importance of documenting immunization status and maintaining immunization cards. Recent efforts by a WHO working group to develop a set of core data for systematic collection will help to harmonize vaccine safety monitoring and meaningful analysis of AEFI data in resource-constrained settings [17].
- Active surveillance of large populations is an important component of vaccine safety monitoring in low-income countries. Study designs and statistical methods should be further refined for these settings. Sentinel site surveillance could be developed for ongoing active safety monitoring, with specific protocols for vaccine-specific adverse events as well as generic adverse events. This sentinel site surveillance should complement work to strengthen passive surveillance systems, which permit signal detection anywhere in a country, and is critical for picking up programmatic errors that may not occur in all sites.
- Vaccine safety training is critical for immunization programs in low-resource settings. WHO has developed new training tools (eg, e-learning tools) and guidance documents to improve country capacity for basic vaccine safety monitoring [22].
- Tools and structures developed and implemented for safety monitoring during a mass vaccination campaign should be sustained for routine activities. Resources available for the campaign provide opportunities for training and tools development. However, resources from the routine immunization budget should also be included in national multiyear plans so vaccine safety is properly monitored throughout the program.
